# Multispectral Plant Disease Detection with Vision Transformer–Convolutional Neural Network Hybrid Approaches

**DOI:** 10.3390/s23208531

**Published:** 2023-10-17

**Authors:** Malithi De Silva, Dane Brown

**Affiliations:** The Department of Computer Science, Rhodes University, Hamilton Building, Prince Alfred Street, Grahamstown 6139, South Africa; d.brown@ru.ac.za

**Keywords:** plant disease identification, deep-learning, CNN, ViT, multispectral images, NIR

## Abstract

Plant diseases pose a critical threat to global agricultural productivity, demanding timely detection for effective crop yield management. Traditional methods for disease identification are laborious and require specialised expertise. Leveraging cutting-edge deep learning algorithms, this study explores innovative approaches to plant disease identification, combining Convolutional Neural Networks (CNNs) and Vision Transformers (ViTs) to enhance accuracy. A multispectral dataset was meticulously collected to facilitate this research using six 50 mm filter filters, covering both the visible and several near-infrared (NIR) wavelengths. Among the models employed, ViT-B16 notably achieved the highest test accuracy, precision, recall, and F1 score across all filters, with averages of 83.3%, 90.1%, 90.75%, and 89.5%, respectively. Furthermore, a comparative analysis highlights the pivotal role of balanced datasets in selecting the appropriate wavelength and deep learning model for robust disease identification. These findings promise to advance crop disease management in real-world agricultural applications and contribute to global food security. The study underscores the significance of machine learning in transforming plant disease diagnostics and encourages further research in this field.

## 1. Introduction

Agriculture plays a crucial role in shaping the global economy, influencing food security, employment, trade, and overall development [1,2]. Most importantly, it is the primary source of food production for the world’s population. As the global population continues to grow, the demand for food increases. The efficiency and productivity of agricultural systems directly impact food availability and security, ensuring that people have access to sufficient and nutritious food [1].

However, agriculture faces numerous threats that can negatively impact crop production, food security, and rural livelihoods. These threats can arise from various sources, including pests and diseases, environmental challenges, and social and political issues [3]. From the above-mentioned threats, plant diseases can significantly negatively impact food production. They affect various crops, including cereals, fruits, vegetables, and other staple foods, leading to reduced yields, quality losses, and economic consequences [4]. Infected plants may produce lower harvests with lower nutritional value, altered taste, or reduced shelf life. Reduced yield can result in food shortages and higher prices for consumers, and low-quality harvests cause farmers to earn less from their crops, impacting their income and economic stability [4,5].

Early identification of plant diseases is a proactive approach to managing crop health and initiating appropriate treatment measures promptly. Timely application of fungicides, pesticides, or other control methods can help to stop the disease from spreading and limit its severity [6]. Therefore, early intervention can save crops from significant damage, ensuring higher yields and preserving the value of the harvest and food safety by reducing the likelihood of contaminated produce reaching consumers [6,7].

Numerous research efforts have been made to tackle the early identification of plant diseases [8]. Machine learning and deep-learning models have become popular non-invasive methods as they have garnered widespread recognition due to their remarkable ability to detect plant diseases in their early stages effectively [9,10].

Existing plant disease datasets are helpful for researchers to experiment and find efficient disease classification strategies. However, dataset collection strategies and environmental factors play a massive role in plant disease identification research.

Mohanty et al. [11] conducted a study using the PlantVillage dataset. Three different variations of the dataset, such as with colour images, grey-scaled images, and images with only leaves segmented, are used to evaluate the impact of the background information on classification. Multiple train–test splits were used to assess the performance from the 80:20 train–test split to the 20:80 train–test split. Two CNN architectures, AlexNet and GoogLeNet, were utilised with transfer learning and training from scratch. The highest accuracies of 99.34% with GoogLeNet and 99.27% with AlexNet were obtained using colour images in the 80:20 train–test split.

In 2017, Brahimi et al. [12] applied shallow and deep learning models on tomato leaf images to identify tomato diseases. They have exacted 14,828 tomato leaf images of nine types of tomato diseases from the PlantVillage dataset. Two deep learning models, AlexNet and GoogLeNet, were trained from scratch and transfer learning for comparison. Transfer learning-based models showed higher accuracies than models from scratch. GoogleNet achieved an accuracy of 99.18% with transfer learning and 97.71% when trained from scratch, while AlexNet yielded 97.35% accuracy when trained from scratch and 98.66% with transfer learning. For shallow models, support vector machine (SVM) and random forest were trained with results of 94.53% and 95.46%. Finally, the authors have proposed that the CNN classifier models with transfer learning perform better than shallow classifiers.

Studies [11,12] combined existing plant disease datasets with machine learning techniques. They showed that deep learning models generally outperform shallow machine learning models. Nevertheless, all of the literature used the PlantVillage dataset, where data were captured under controlled environmental conditions. Hence, the same results may not have been obtained if images outside that particular environmental condition were given.

To address the constraints associated with utilising publicly accessible datasets for detecting plant diseases, numerous researchers [13,14] have attempted to create datasets that show plants in their native surroundings, including complex backgrounds.

In 2020, Ramesh and Vydeki [13] performed a study to identify healthy and diseased rice plants. Six hundred fifty images were captured using a digital camera for four disease categories: bacterial blight, brown spot, sheath rot, and blast. The dataset was first resized and then removed from the background of RGB images, leaving only the leaf part of the images. The diseased portion is extracted from the leaf images using K-means clustering, and then texture and colour features are extracted. The classification was performed with an optimised Deep Neural Network, and results were compared with existing classifiers such as Artificial Neural Network (ANN) and Deep AutoEncoder (DAE). The proposed classification model overperformed existing models with high accuracy of 98.9%, 95.7%, 92%, and 94% for the blast, bacterial blight, sheath rot, and brown spot, respectively.

A hybrid vision transformer model to detect apple diseases was proposed by Li and Li [14] in 2022. Five apple foliar disease images were collected using Apple and Android mobile phones. A total of 2029 images were collected in three different Apple stations to improve the diversity of the dataset. The dataset was extended to 15,834, using image pre-processing techniques such as rotation, horizontal and vertical mirroring, and Gaussian blurring. The work proposed a new model called ConvViT. The new model performance was compared with CNNs such as MobileNetV3, VGG-16, EffientNetB0, and Resnet-18, and Transformer models Deit-tiny, Swin-tiny, and ViT-small networks. The results showed that the Swin-tiny model obtained the highest 96.94%, followed by the proposed model with 96.85%.

Studies [13,14] employed newly acquired datasets from natural settings. Nevertheless, all the datasets contained Red, Green, Blue (RGB) images. RGB images solely encompass the visible segment of the electromagnetic (EM) spectrum, and similarly to the human eye, the visible spectrum is ineffective at detecting early disease [15]. When visible symptoms are apparent, plants might have already surrendered to infection, potentially leading to widespread disease propagation.

In 2022, a study by De Silva and Brown [16] involved capturing images from ten distinct agricultural plant species in the natural environment. These images were acquired using a Canon EOS 800D camera, Kolari Vision, Raritan, NJ, USA equipped with a Hot Mirror filter for capturing RGB images and a K665 filter for NIR images in the 665–1000 nm range. The research encompassed the comparison of two datasets, employing CNN models, namely MobileNet, ResNet-50V2, VGG-16, and GoogleNet. Notably, the ResNet-50V2 model exhibited superior performance over other models for both RGB and NIR datasets, achieving test accuracies of 98.35% and 94.01%, respectively. Furthermore, within the same context [17], the authors utilised the identical dataset excluding RGB data to evaluate eight distinct CNN models across various train–test split ratios ranging from a minimum of 10:90 to a maximum of 90:10. The study’s conclusion highlighted that the Xception model surpassed the other seven models, demonstrating perfect (100%) training and testing accuracies in the 80:20 standard train–test split configuration.

Studies [16,17] compare disease classification results obtained using a single NIR and RGB filters. In theory, NIR images contain a broader information spectrum, encompassing wavelengths beyond the visible range [18]. Therefore, higher accuracy should be reported with NIR images. However, the authors concluded that RGB images in natural environments provided better accuracies than NIR images. This intriguing outcome prompted the selection of multiple filters, each tuned to capture distinct wavelength ranges. This aimed to comprehensively compare the results obtained from these diverse filters and those derived from RGB images.

De Silva and Brown [19] captured a new multispectral dataset using a Canon EOS 800D camera with four distinct filters (BlueIR, K590, Hot Mirror, and K850). The dataset contained images of five fruit plants, namely avocados, tomatoes, limes, passion fruit, and gooseberries, collected under various weather conditions with temperatures spanning from 22 to 37 °C. The datasets had varying numbers; BlueIR had 648 images, K590 had 369 images, Hot Mirror had 890 images, and K850 had 1109 images. Three well-known CNN models, namely Xception, DenseNet-121, and ResNet-50V2, were used to investigate the optimal CNN model for the newly acquired dataset. The highest test accuracies were achieved using DenseNet-121 for all datasets except the Hot Mirror, and the K850 was the best-performing filter with a test accuracy of 86.16%. During the data collection, data sets for some filters were collected on sunny days with clear skies with temperatures of 25–28 °C. At the same time, rain and minimal sunlight affected some filters, and images had water drops on the leaves. Therefore, the authors concluded that weather conditions and natural backgrounds influenced the outcomes of the tested CNN models.

The same dataset used in [19] was subject to testing in [20] utilising prominent ViT models such as ViT-S16, ViT-B16, ViT-L16, and ViT-B32. ViT-B16 emerged with superior training and testing results with transfer learning, outperforming the other ViT models. The authors recommended ViT-B16, especially when constrained computational resources and compact models are preferred.

The evaluation in Brown and De Silva [21] took a different approach, employing hybrid vision transformers ViT_R26_S32 and ViT_R50_L32 on the same multispectral dataset in [19]. The authors identified the highest test accuracy with ViT_R26_S32 (88.17%) on the K850 filter. Comparisons were drawn between the outcomes achieved with CNN models in [19], ViT models in [20], and hybrid ViT models. They found that ViT-B16 performed the best in test accuracy with the BlueIR filter, followed by ViT_R26_S32 with the K850 filter. Notably, the K850 dataset showcased the most favourable performance when utilising CNN and hybrid ViT models, whereas the BlueIR dataset demonstrated the highest performance when using ViT models alone. Across all the models scrutinised in [19,20,21], the K590 dataset consistently yielded the least satisfactory results. As a future direction, all these papers suggested collecting data under uniform environmental and climatic conditions to replicate experiments and validate the robustness of the findings.

The primary limitation found in the datasets utilised in [19,20,21] lies in the unequal dataset sizes across different filters. The distribution of images across various classes is also uneven, as it was collected in a natural setting. These studies collectively concluded that the K590 filter yielded the lowest accuracy, possibly attributed to the relatively small dataset size rather than a filter-specific issue. As a result, meaningful conclusions regarding the efficacy and dependability of the filters can only be drawn when comparable dataset sizes are used for all filters.

Hence, this paper aims to comprehensively investigate early plant disease identification using a newly acquired multispectral dataset comprising six filters, all captured in natural environmental settings under consistent conditions with balance classes. This research compares various environmental conditions, dataset sizes, and class balance considerations, both for the newly acquired dataset and the unbalanced dataset used in [19,20,21], to check the impact of these factors on the research outcomes.

The remainder of this paper is organised as follows: Section 2 describes the materials and methodology used, and Section 3 presents the results of the experiments. Discussion of the results is in Section 4, and Section 5 concludes the paper by summarising the main findings and future directions.

## 2. Materials and Methods

### 2.1. Data Collection

**Figure 1:**
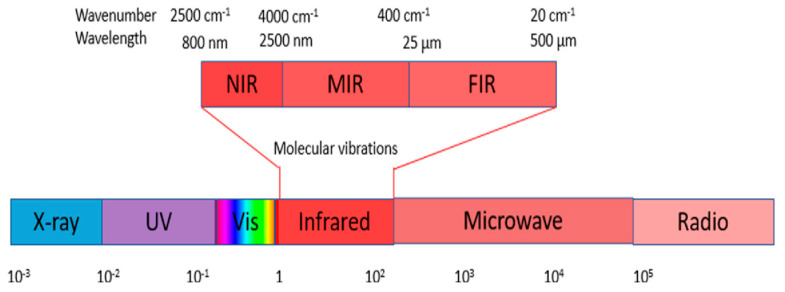
The Infrared (IR) wavelength range: A spectrum encompassing Near Infrared (NIR), Mid Infrared (MIR), and Far Infrared (FIR) [22].

**Figure 2:**
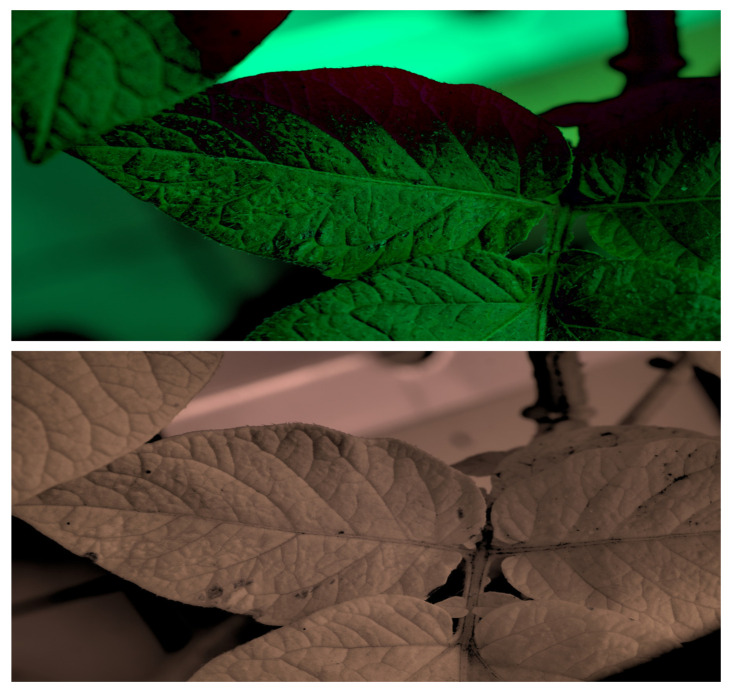
Comparison of leaf images captured using RGB and NIR filters to assess the suitability of these filters in revealing early disease symptoms in plants.

Figure 1 shows that NIR comprises wavelengths beyond the visible range. As shown in Figure 2, NIR images hold crucial insights not available in the visible spectrum, enabling the identification of abnormal conditions beyond the limits of human sight. Therefore, NIR images provide valuable information that supports early intervention strategies in agriculture, enabling proactive disease management and improving crop yields.

NIR images can be captured using hyperspectral and multispectral cameras. Hyperspectral cameras use specialised sensors to capture data in many narrow and closely spaced spectral bands, often spanning from the ultraviolet to the infrared regions of the EM spectrum. This fine spectral resolution enables the identification and discrimination of materials and substances based on their unique spectral signatures [23]. Managing hyperspectral images requires specialised knowledge, dedicated hardware, and software. These images are notably large, and only some captured spectral bands contribute crucial insights into plant diseases. As a result, in disease identification research, scientists often refine their analysis by selecting specific spectral bands that carry the most relevant information [15].

Unlike hyperspectral images, which capture data in numerous narrow and contiguous spectral bands, multispectral images capture data in a limited number of broader spectral bands [24]. This approach provides less detailed spectral information than hyperspectral imaging, but is often more practical and cost-effective. Multispectral imaging is more common and accessible due to its lower data acquisition and processing requirements. While it provides less detailed spectral information, it still offers valuable insights for a wide range of applications where the finer spectral resolution may not be necessary [25].

This study gathered data utilising a customised digital single-filter reflex (DSLR) camera, which has the capability to capture various segments of the EM spectrum depending on the filter employed. A Canon EOS 800D camera was chosen due to its cost-effectiveness, being significantly more affordable, approximately 20 times less expensive than a multispectral camera and 100 times less expensive than a hyperspectral camera. It is flexible for capturing standard and specialised images with user-friendly interfaces and supports a wide range of filters readily available on the market that enable capturing multispectral bands.

Six filters were used for data collection, and each filter allows only limited ranges encompassing the visible and near-infrared spectrum, as shown in Table 1. The K590 filter captures the 590–1000 nm range, while K665, K720, and K850 allow only 665–1000 nm, 720–1000 nm, and 850–1000 nm wavelengths, respectively. BlueIR allows both blue (450–500 nm) and IR (800–1000 nm) ranges when capturing images. The Hot Mirror filter cuts all the wavelengths except the visible portion of the EM spectrum, allowing it to capture RGB images.

Data collection involved selecting three plant species from different varieties of plant types. Tomato, potato and papaya were chosen for experiments, as all three plants are susceptible to fungal diseases such as blights, rots, and mildews and viral infections with similar symptoms of leaf spots, wilting, discolouration, decay, yellowing, and mosaic patterns on leaves [26]. Diseases on plant leaves were mainly focused on when leaves were damaged. This directly affects the photosynthesis process and degrades the amount and the quality of the harvest [27].

Six identical images were captured per sample for each plant, maintaining consistent factors such as angle, zoom, and lighting covering all the filters. Samples of the captured images are depicted in Table 2. Each image contained at least one leaf and included a natural background. Throughout the image capture process, efforts were made to maintain stable environmental conditions across images captured in six filters. The data collection took place during winter 2023 (June and July) between 8:00 a.m. and 1:00 p.m., with temperature variations recorded between 17 °C and 20 °C. Soil pH and moisture levels were maintained within the range of 6.5 to 7.0, while natural sunlight served as the primary light source.

Table 3 shows the compositions of captured data for one filter, which contains 442 images each. Altogether, the new balanced dataset contains 2652 images, covering six filters.

### 2.2. Data Preprocessing

Rawtherapee software, version: 5.8-3 (www.rawtherapee.com) which incorporates the Retinex algorithm [28], was used to process raw images in this study. Rawtherapee stands out as a robust open-source application designed for efficiently enhancing raw images while ensuring optimal performance [29]. The Retinex theory explains the mechanisms within the human visual system responsible for preserving colour consistency, offering a means to estimate and standardise image illumination [30]. This theory uses physiological and psychological assumptions to explain colour perception across varying lighting conditions. This processing technique substantially reduced the average size of raw images, decreasing from 25 MB to approximately 200 KB. Utilising the Retinex algorithm, the following parameters were employed: Scale = 3, Slope = 3, and Colour Restoration Factor = 1.3. Moreover, Dynamic Range Compression Parameters were set with Highlight Tonal Width = 80 and Shadow Tonal Width = 80. Samples of the processed images are shown in Table 2. Converted images were of the size of 899×600, and all the images were resized to 224×224 for the deep learning models.

Given that the dataset utilised for this study comprised approximately 2600 images, and considering the data-intensive nature of deep learning models, the application of data augmentation is needed to enhance the diversity and size of the training dataset. Augmentation methodology aimed to support the model’s capacity for generalisation and resilience by generating novel instances by implementing a spectrum of transformations on the existing data, including adjustments in lighting conditions, perspectives, scales, and other relevant attributes [31]. Augmentation strategy mitigates overfitting tendencies, amplifying the model’s aptitude for detecting patterns within novel, unobserved data.

While incorporating data augmentation techniques can undoubtedly enhance the efficacy of machine learning models by diversifying the training data, it is crucial to recognise that an excessive augmentation application might inadvertently introduce distortions into the dataset. Consequently, this study employed specific augmentation measures as shown in Table 4.

For the shear and zoom range, 0.2 is commonly used as an overly high range may result in warped or pixelated images that do not effectively represent realistic variants of the original data. Augmentations relating to brightness, hue, contrast, and saturation were intentionally omitted, as the environmental conditions were consistently maintained across all images captured through the six filters. Similarly, cropping and horizontal and vertical shifting were not used due to the variability in the positioning of plant leaves within the images, thereby preserving crucial information.

### 2.3. Feature Extraction and Classification

Feature extraction plays a pivotal role in classification, as it involves recognising and isolating the prominent attributes from the unprocessed data. These extracted features subsequently serve as the foundation for differentiation among distinct classes.

This work aims to reproduce experiments conducted in previous studies [19,20,21], employing a harmonised dataset for all filters and comprehensively assessing whether analogous conclusions can be drawn.

In this study, the deep learning models were all employed with transfer learning, which involves using an existing model to initialise the weight parameters of a target model. In this research context, the CNN, ViT, and hybrid ViT models were initially pre-trained using the ImageNet dataset due to the limited size of the dataset. With transfer learning, pre-trained models are empowered to overcome the limitations of scarce data. It effectively reduces extended training times and enhances overall performance and interpretability.

#### 2.3.1. Convolutional Neural Network (CNN) Models

CNN models consist of interconnected layers, each performing specific operations that progressively extract and abstract features from raw data. The fundamental components of CNNs are convolutional layers, pooling layers, and fully connected layers [32]. Convolutional layers use filters to slide over the input data, extracting relevant features by performing convolutions. These filters detect edges, textures, and other essential characteristics present in the data. The subsequent pooling layers reduce the spatial dimensions of the data, focusing on preserving the most salient features while discarding redundant information. The final layers of a CNN often involve fully connected layers, which consolidate the extracted features and make predictions based on them. The hierarchical nature of CNN architecture enables the models to capture both local and global patterns, making them highly effective for tasks like image recognition, object detection, and even more intricate tasks. This work used CNN models, Xception [33], ResNet-50V2 [34] and DenseNet-121 [35], pre-trained on ImageNet for the experiments.

#### 2.3.2. Vision Transformer (ViT) Models

**Figure 3:**
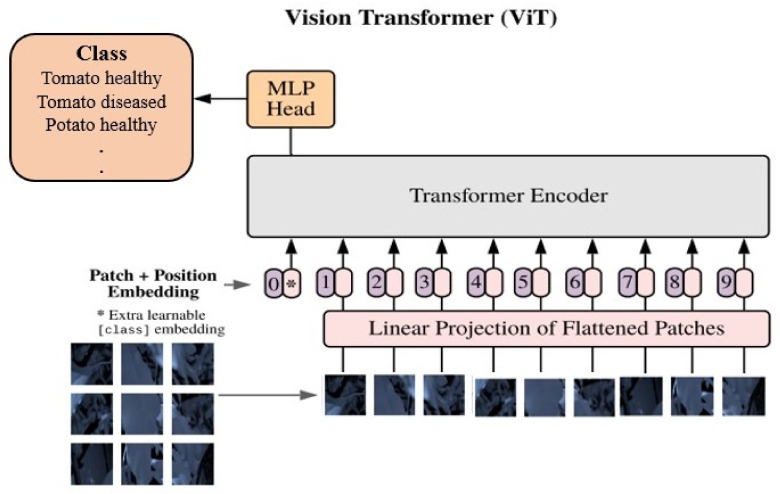
Key components of the (ViT) architecture from bottom to top: Image Patch Division, Linear and Position Embeddings with a learnable token, Transformer Encoder, and the multi-layer perceptron head for classification.

As Figure 3 depicts, ViT models revolutionise image analysis by treating images as sequences of patches. The core of a ViT model is the self-attention mechanism [36], which allows the model to weigh the importance of different patches in relation to each other, effectively capturing long-range dependencies and relationships within the image. In a ViT model, the input image is divided into fixed-size patches, which are then linearly embedded and transformed into sequences of vectors. The self-attention mechanism subsequently processes these sequences, enabling the model to learn contextual relationships between different patches. Positional encodings are added to the embeddings to preserve spatial information [37]. ViT models are scalable and can be trained on large datasets and extended to various input sizes, making them adaptable to different applications.

This work evaluates the ViT-S16, ViT-B16, and ViT-L16 models [20] with transfer learning. Within the ViT architecture, the prefixes “S”, “B”, and “L” indicate the size of the model, which is determined by both the number of transformer layers and the hidden size of the self-attention mechanism. More precisely, “S” designates a small model, “B” represents a base model, and “L” signifies a large model. For the conducted experiments, this research employed the following configurations: a small model comprising eight transformer layers with a hidden size of 384, a base model comprising 12 transformer layers with a hidden size of 768, and a large model comprising 24 transformer layers with a hidden size of 1024. All models used 16 × 16 patch size.

#### 2.3.3. Hybrid ViT Models

**Figure 4:**
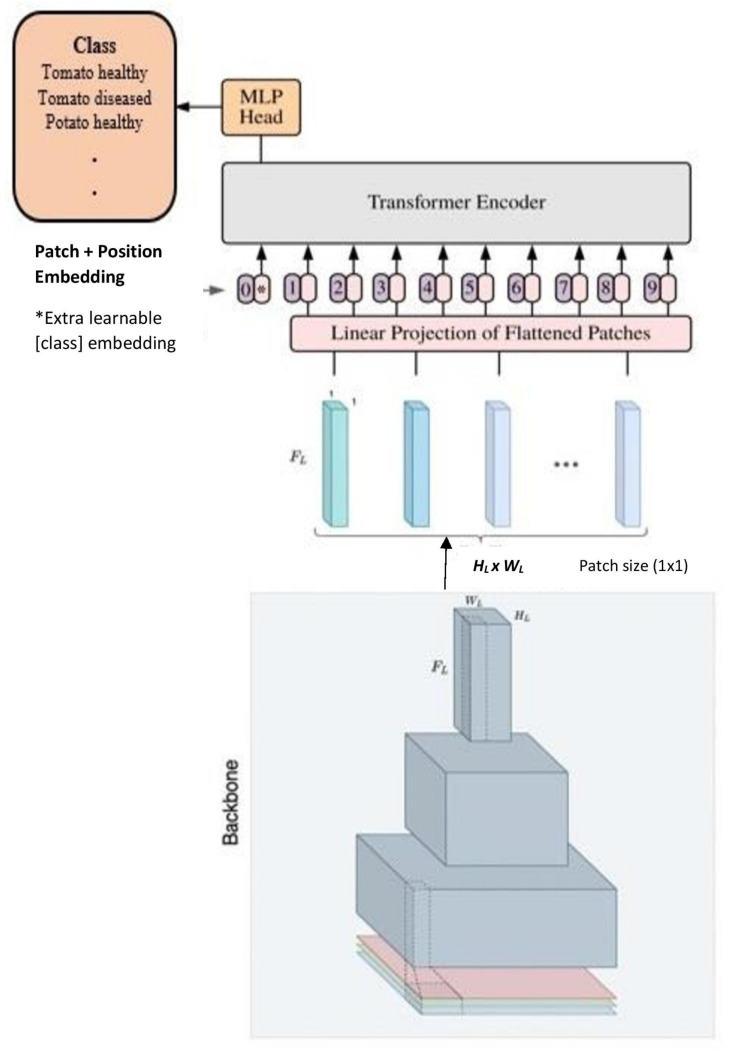
Hybrid ViT architecture: a pre-trained CNN backbone takes the input image and captures hierarchical image features with output features transferred to a pre-trained ViT model for classification.

Hybrid ViT extends the ViT model architecture by merging the capabilities of CNNs and transformers for visual recognition tasks [38]. In this approach, the initial step involves inputting an image into the hybrid model, which then processes the image through the CNN component, resulting in a feature map. This feature map is subsequently partitioned into patches, flattened, and provided as input to the ViT model, as depicted in Figure 4. The ViT model takes in these patches as a sequence of tokens and applies a series of transformer blocks to encode spatial and channel-wise relationships among the patches. The encoded sequence is channelled through a linear classifier, culminating in the generation of final predictions. This study utilises base models ResNet-26 and ResNet-50 alongside ViT-S32 and ViT-L32 as the ViT model variations.

In the ViT_R26_S32 model, the input image undergoes partitioning into non-overlapping patches, each of size 32×32 pixels. These patches are subsequently subjected to processing by the ResNet-26 backbone, extracting local features. To enable the model to capture long-range dependencies, a Transformer-based head is employed for global self-attention across the extracted features. The notation ‘S’ in ViT_R26_S32 denotes a smaller iteration of the original ViT architecture, comprising just eight Transformer layers [38]. The ViT_R50_L32 uses a ResNet-50 backbone for local feature extraction and a Transformer-based head for global self-attention. ViT model uses a 32×32 pixel patch size, and the ‘l’ prefix is designated for the ViT_larger model, incorporating 24 transformer layers [38].

#### 2.3.4. Swin Transformer Models

The Swin Transformer introduces a hierarchical approach to handling self-attention computation. This hierarchical design is unlike ViTs, as it efficiently scales the model to larger input image sizes without significantly increasing computational complexity [39]. The key innovation lies in the concept of shifted windows and the partitioning of images into non-overlapping local windows at different scales. This work examines the performance of Swin_Tiny, Swin_Small, Swin_Base and Swin_large models. All the models work with a patch size 4×4 and an attention window size of 7 [39].

### 2.4. Experimental Setup

Experiments were conducted on a server with an AMD Ryzen™ 9 5950X 4.9 GHz 16-core CPU combining G.Skill Ripjaws V 64GB RAM and NVIDIA RTX 3090 GPU.

All models integrated dropout layers into their architecture to support generalisation and control overfitting. These layers selectively deactivate units during training, mitigating the risk of the model excessively tailoring itself to the training dataset. The Adam optimiser was employed, utilising a batch size of 32 and initialising the learning rate to $1\times 10^{-4}$, beta_1 = 0.9, beta_2 = 0.999, epsilon = $1\times 10^{-7}$ for optimisation. Initially, 300 epochs were used, and the early stopping strategy was adopted to prevent overfitting. This approach constantly evaluates the validation loss and terminates training if this metric fails to decrease over a specified number of epochs, which is 15 epochs in the experiments conducted in this paper. To mitigate overfitting, the restore_best_weights parameter is configured as True, so the model’s weights are reinstated to their optimal state when early stopping is activated, effectively returning to the model’s peak performance.

### 2.5. Evaluation

At first, the dataset was split into three subsets: the training set, the validation set, and the testing set, using an 80:10:10 ratio. The model was then trained using the training set, evaluated and fine-tuned during the training process with the validation set, and its predictions were assessed using the testing set. The test set is unused during training and only evaluated at the end during inference.

To comprehensively assess the models’ performance, this study employs a confusion matrix along with metrics including accuracy, precision, recall, and F1 score, as illustrated by Equations (1)–(4):

(1) $$Accuracy=\frac{TP+TN}{TP+TN+FP+FN}$$

(2) $$Precision=\frac{TP}{TP+FP}$$

(3) $$Recall=\frac{TP}{TP+FN}$$

(4) $$F1=\frac{2*Precision*Recall}{Precision+Recall}=\frac{2*TP}{2*TP+FP+FN}$$

where:

- TP represents true positive samples;
- TN represents true negative samples;
- FN corresponds to false negative samples;
- FP signifies false positive samples.

Accuracy assesses the classifier’s ability to measure overall correctness or the proportion of correctly predicted instances from the total instances in a dataset. Precision quantifies the ratio of correctly identified positive cases out of all positive cases determined by the classifier, recall computes the proportion of predicted positive points relative to the total positive cases, and the F1 score provides a balanced measure by combining precision and recall. It is calculated as the average of these metrics across multiple classes.

## 3. Results

As the accuracy values for Swin Transformer models with the unbalanced dataset used in [19,20,21] are not available in the literature, experiments were conducted to obtain accuracies. As per Table 5, the BlueIR filter dataset consistently leads to higher accuracy across all model variants, followed by the K850 dataset. From Swin models, the Swin_large model showed the highest test accuracy. Hence, the filter choice impacts accuracy, but the impact varies depending on the model size.

Table 6 shows the testing accuracies obtained with the new balanced dataset from the experiments using different models for six filters. Based on the outcomes observed, the ViT and hybrid models are comparable in most cases to CNNs and outperformed by DenseNet-121 when prioritising K720. Notably, the ViT-B16 model consistently attained an accuracy of 82–84%, showing higher performance with most of the filters.

Among the CNN models, Xception and DenseNet-121 demonstrated relatively comparable performances. In contrast, ResNet-50V2 displayed the lowest accuracy across all evaluated models, averaging around 75%. However, a significant improvement in accuracy was achieved by the hybrid model that combined ResNet-50V2 with a ViT model, yielding an average accuracy of approximately 82%. The model’s depth or size did not significantly impact accuracy, as smaller models occasionally exhibited higher accuracy than their larger counterparts.

Based on Table 6, when analysing the performance for the utilised filters, K590, K665, and K720 displayed similar accuracies among all deep learning models, marginally favouring the K590 filter. This trend holds in analysing the performance of individual CNN, ViT, hybrid ViT, or Swin Transformer models used in this work. Interestingly, CNN models exhibited lower performance with the K850 dataset, while Swin Transformer models demonstrated their lowest accuracy with the BlueIR and Hot Mirror datasets.

Since the K50, K665, and K720 filters exhibited comparable accuracy to the various deep learning models tested, the models’ speeds were evaluated by measuring the time required to complete a single epoch. Across all three filters, most models exhibited a similar step time, approximately 2 s per epoch. However, the ViT_L16 and Swin_large models stood out with a slightly longer execution time of 3 s per epoch.

**Figure 5:**
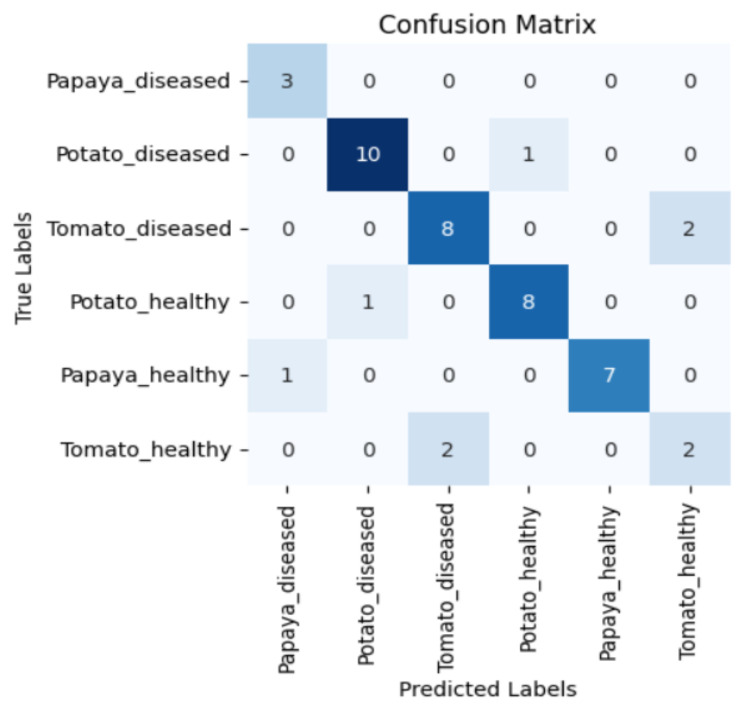
Confusion matrix for ViT-B16 model with the K665 filter.

**Figure 6:**
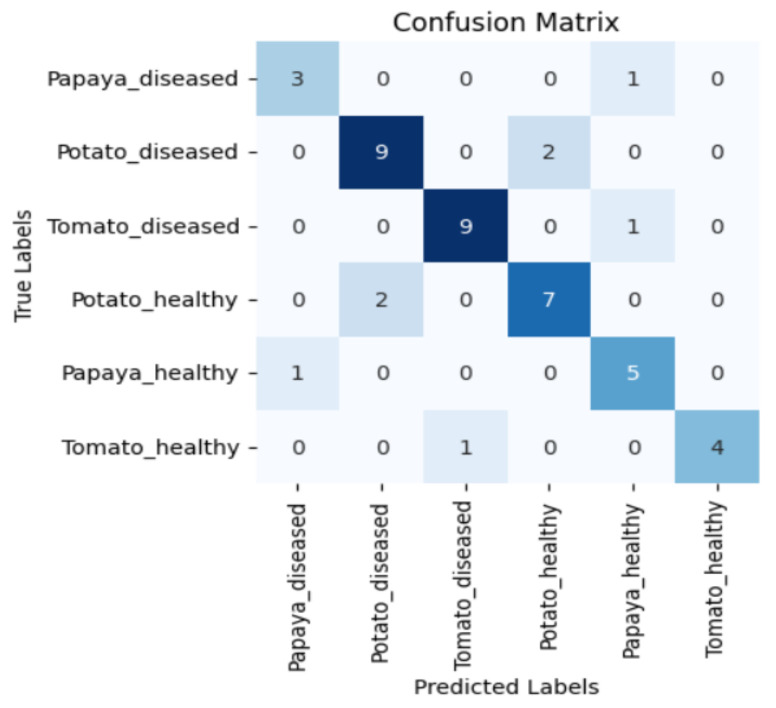
Confusion matrix for ViT_R50_L32 model with the K720 filter.

**Figure 7:**
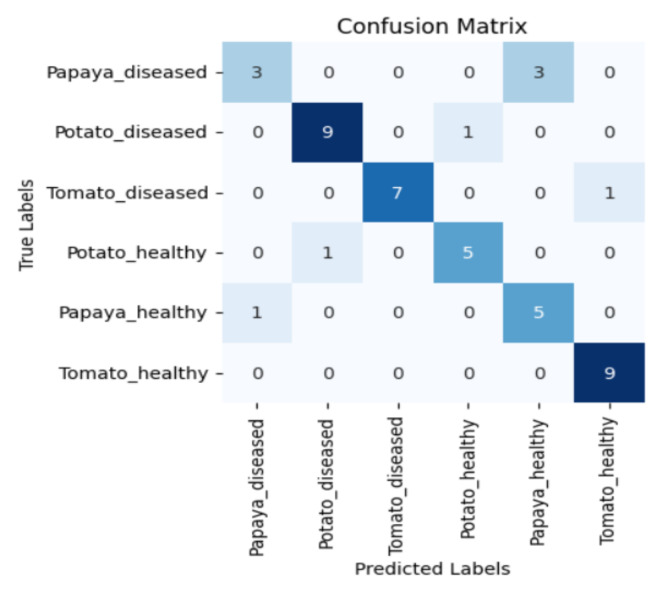
Confusion matrix for Swin_base model with the K850 filter.

**Figure 8:**
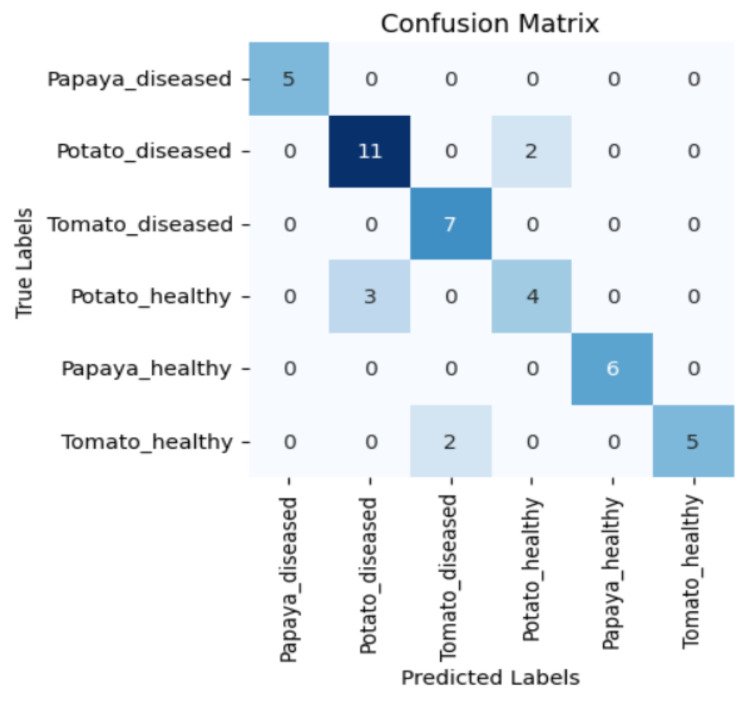
Confusion matrix for Xception model with the Hot Mirror filter.

Figure 5, Figure 6, Figure 7 and Figure 8 display the confusion matrices encompassing four distinct deep learning categories: CNNs, ViTs, hybrid ViTs, and Swin Transformers. These models were selected as they showed a similar accuracy rate of approximately 84%, encompassing filters capable of capturing both visible and NIR spectra and those focusing solely on visible or NIR. As indicated by the findings presented in Figure 5, Figure 6, Figure 7 and Figure 8, when utilising the new balanced dataset, a common trend emerged wherein all plant species experience misclassifications, erroneously categorising diseased plants as healthy and vice versa. Notably, there is a higher tendency to classify diseased plants as healthy than the reverse scenario.

**Figure 9:**
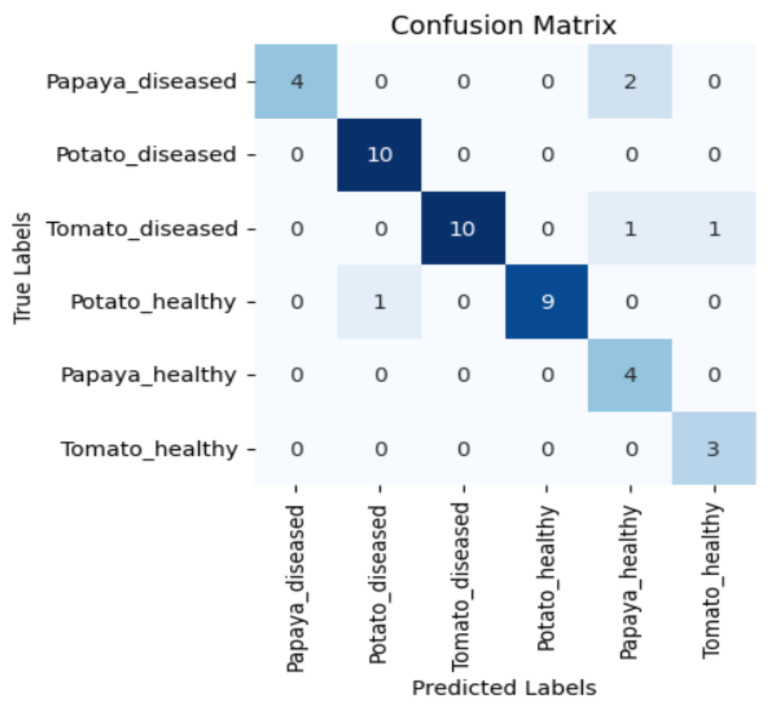
Confusion matrix for DenseNet-121 model with the K720 filter.

**Figure 10:**
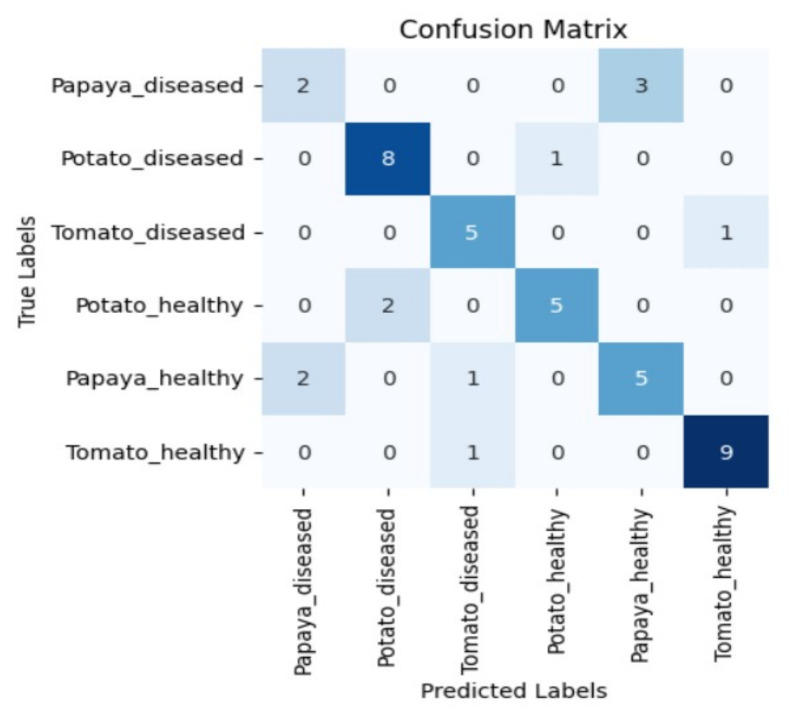
Confusion matrix for Swin_small model with the K850 filter.

Moreover, in certain instances, as evidenced by the confusion matrices in Figure 9 and Figure 10, there were instances of misclassification among different plant species. This misclassification was particularly prevalent between papaya and tomato species. The unique leaf shapes of these plants may have contributed to this misclassification, especially when capturing images from various angles, where certain angles could capture portions of a papaya leaf that resemble tomato leaves.

Tomato is the common species for both the unbalanced and the new balanced datasets. The composition of healthy and diseased tomato data of the two datasets are shown in Table 7.

Hence, for comparison purposes for both datasets, precision, recall, and F1 score of healthy and diseased tomatoes are tabulated in Table 8, Table 9, Table 10 and Table 11, Table A1 and Table A2. In the previous literature, Swin Transformer models were not investigated with the unbalanced dataset. Hence, in this work, Swin Transformer models have also been experimented with, and findings are shown in Table 9, Table 11 and Table A2.

Precision is a metric that gauges the reliability of a model’s positive predictions. As Table 8 shows, regarding various models and filters, the precision for identifying healthy tomato leaves tends to surpass that for diseased leaves. Overall, precision associated with diseased leaves exhibits significant fluctuations, indicating that the model’s proficiency in disease detection can diverge based on the specific filter and model employed.

According to the results in Table 8, the K665 showcases the most elevated precision for healthy leaves, averaging 90.7%. In contrast, the K720 filter dataset achieves the most precise positive predictions for identifying afflicted plants, with an average precision of 93.8%. The K720 filter also attains the highest average precision for healthy and diseased plants.

Taking the performance of the deep learning models into account in Table 8, ViT-B16 stands out with the highest average precision of 95.7% for identifying healthy leaves. At the same time, DenseNet-121 exhibits the highest average precision of 89.2% for identifying diseased plants. In the broader picture, ViT-B16 garners an overall average precision of 90.2% for accurately identifying healthy and diseased plants.

As demonstrated in the findings presented in Table 8, a similar pattern emerges in Table 9, where the precision in distinguishing healthy leaves tends to surpass that in identifying diseased leaves. As Table 9 showcases, the K720 filter dataset exhibits the highest precision for healthy and diseased leaves. When evaluating the models’ performance, ViT-B16 takes the lead with an average precision of 92.5% in identifying healthy leaves. Moreover, it maintains a notable average precision for accurately discerning healthy and diseased plants.

Recall is an important metric in situations where identifying positive cases is crucial. A higher recall value indicates that the model better identifies all relevant positive instances, minimising the chances of missing potentially important instances.

The recall findings in Table 10 reflect a pattern similar to that observed in the precision analysis for the new balanced dataset. Specifically, the K665 filter displayed the highest average recall value for identifying healthy plant leaves, while the K720 filter demonstrated superior recall rates for identifying diseased plant leaves. Notably, the K720 filter emerged as the leading performer when identifying positive healthy and diseased leaves. Remarkably, the ViT-B16 model consistently delivered elevated recall values across all filters. It notably achieved the highest recall value for correctly identifying diseased plant leaves, culminating in an overall average recall of an impressive 90.8% for healthy and diseased plants.

Table 11 shows the recall values obtained with the unbalanced dataset. The Hot Mirror filter dataset emerges as the standout performer, showcasing the highest recall values for healthy and diseased tomato leaves. Leading the way, ViT-B16 obtained a high average recall in detecting healthy leaves. Furthermore, it maintains a remarkable average recall score, adeptly distinguishing between healthy and diseased plants.

The performance metrics, including precision in Table 8, recall in Table 10, and F1 score in Table A1, extracted from the novel multispectral dataset displayed a consistent pattern for both the optimal filter and the deep learning model. The K665 demonstrated the highest metrics for accurately identifying healthy plant specimens; the K720 filter dataset provided top-tier predictions for recognising diseased plants. Overall, the K720 filter emerged as the superior performer among all filters. Regarding model performance, ViT-B16 consistently demonstrated the highest performance metric values in distinguishing between healthy and diseased leaves.

Furthermore, the unbalanced dataset showcased a harmonious trend across all matrices. The Hot Mirror filter was exceptional across all matrices, distinguishing healthy and diseased leaves. Regarding precision in Table 9, recall in Table 11, and F1 score in Table A2, ViT-B16 consistently achieved the highest performance, mirroring the outcomes observed with the new balanced dataset.

## 4. Discussion

This paper examines the impact of balanced and unbalanced multispectral datasets, the effectiveness of filters, and the most suitable deep learning model in early plant disease identification research.

Due to the unbalanced nature of the dataset used in the previous literature, findings in [19] for CNN models, [20] for ViT models, and [21] for hybrid ViT models, along with Swin Transformer models depicted in Table 5, consistently highlighted superior accuracies of the K850 and the BlueIR filters. The new balanced dataset mitigates discrepancies in these outcomes. Nonetheless, the K590 filter that demonstrated the least favourable performance in [19,20,21] is notably ranked as the top performer in the new balanced dataset. Hence, the conclusions made in [19,20,21] indicating that the K590 filter was the least effective performer are rendered invalid by the accuracy values obtained from the newly balanced dataset in Table 6. In the unbalanced dataset, this discrepancy could be attributed to the dataset’s size, with the K850 dataset containing a substantial 1109 images, whereas the K590 dataset is considerably smaller, comprising only 369 images [19].

Tomato is the common species for both datasets, prompting an evaluation of filter performance concerning precision, recall, and F1 scores. As depicted by Table 7, it becomes evident that the new dataset uniformly presents 148 instances of both healthy and diseased data for each filter. However, the unbalanced dataset has varying healthy and diseased plant data counts, ranging from 222 instances (K850) to 47 instances (K590) [19].

Within the new balanced dataset, results obtained for precision in Table 8, recall in Table 10, and F1 score in Table A1, the K665 filter best classified healthy plant samples. In contrast, the K720 filter excelled in accurately identifying diseased plants. The K720 filter emerged as the frontrunner regarding overall performance among all filters. Following closely, the K590 and BlueIR filters also sustained commendable performance, whereas the K850 and Hot Mirror filters demonstrated comparatively lower efficacy. Considering the accuracy of the new dataset, it is evident that the same pattern was observed concerning filters. Since all datasets were intentionally balanced and environmental conditions were consistently upheld during the data capture process, variations in results are solely attributed to the filters utilised and the wavelengths they captured.

However, based on the precision in Table 9, recall in Table 11, and F1 score in Table A2 derived from the unbalanced dataset, the Hot Mirror filter exhibited the highest performance, closely followed by the K850 filter. Conversely, the K590 filter consistently showed the lowest performance across all performance metrics. The dataset’s size influences these outcomes [19] and the specific environmental conditions during data acquisition. Consequently, when comparing the results obtained with the new balanced dataset, it is important to note that the results obtained with the unbalanced dataset might not definitively reflect the actual filter’s performance due to influencing factors [21].

Considering the accuracy, precision, recall, and F1 score discussed earlier for the new balanced dataset, it becomes apparent that filters encompassing both the visible and near-infrared segments (Table 1) of the spectrum yield improved results in identifying plant diseases. Conversely, filters exclusively targeting the visible (Hot Mirror) or NIR (K850) portions may exhibit comparatively lower performance.

Overall, the accuracy achieved by various deep learning models across the two datasets shows that the same models consistently exhibited the highest accuracy for each type of deep learning architecture. In the context of the unbalanced dataset, referring to the findings of [19] for CNN models, [20] for ViT models, [21] for hybrid ViT models, and also for Swin Transformer models in Table 5, DenseNet-121, ViT-B16, ViT_R26_S32, and Swin_large demonstrated the most impressive performances. These results hold true when applied to the newly introduced balanced multispectral dataset. Across both datasets, when evaluating precision, recall, and F1 scores for CNNs, ViTs, and hybrid ViTs, the models that exhibited superior accuracy also demonstrated the top performance.

Considering accuracy across all species and evaluating precision, recall, and F1 scores specifically for tomatoes, the ViT-B16 model consistently exhibited exceptional performance [21], and the ResNet-50V2 model always displayed the least favourable performance for both unbalanced [19] and balanced datasets. Across all assessment metrics, a consistent trend emerged, highlighting the superior overall performance of ViT models, followed by CNN models (excluding ResNet-50V2).

Both datasets are constrained in data availability, comprising a limited number of images, with 3016 images for the unbalanced dataset and 2652 images for the new balanced dataset. As a result, the performance of larger models, such as ViT-L16 (≈305 M parameters) and ViT_R50_L32 (≈330 M parameters), did not exhibit any enhancement across any evaluation metric despite their increased size [20,21]. Likewise, smaller models, including ResNet-50V2, ViT-S16, and Swin_tiny, characterised by a range of total parameters between 24 M and 29 M, failed to generalise effectively with either of the datasets [19,20].

Small models might have high bias and low capacity, making them unable to capture the underlying patterns in the data, even if it is a small dataset [21]. On the other hand, larger models might have too much capacity and can overfit, especially with limited data. Moderate-sized models strike a balance, offering sufficient capacity to capture patterns without overfitting. Further, small models might not have enough parameters to learn complex features from the data, while larger models can memorise noise [21].

Models with a moderate number of parameters, like ViT-B16, are better equipped to learn important features indicative of the underlying relationships in the small dataset [21]. These models can generalise better than small models, which might underfit, and larger models, which might overfit. The balance in moderate-sized models allows them to generalise more effectively.

## 5. Conclusions

This research explored the outcomes of balanced and unbalanced multispectral datasets collected in a natural environment. This extensively evaluated cutting-edge deep learning models, including Convolutional Neural Networks (CNN), Vision Transformer (ViT), and hybrid models, aiming to determine the most effective model for plant disease detection. Comparative analyses are conducted between the results obtained from the new balanced and unbalanced multispectral datasets to identify the optimal deep learning model for advancing early disease identification research. The outcomes consistently demonstrated the superiority of ViT models, especially ViT-B16.

Moreover, this study exploited the applicability of various filters in early plant disease identification research, ultimately pinpointing the most appropriate imaging techniques. The findings revealed that filters capable of capturing both visible and NIR spectra (i.e., K590, K665, and K720) performed better in disease identification than filters limited to visible or NIR spectra (i.e., Hot Mirror, K850) alone.

Additionally, the research delves into comparisons across dataset sizes and class balance considerations, both for the new and unbalanced datasets. It confirmed that these factors have an impact on the research outcomes.

Uniform class sizes and consistent environmental conditions across various filters with the new balanced dataset highlighted the influence of distinct environmental factors, and varying class sizes significantly impact the outcomes. Erroneous conclusions regarding K590 being the least effective and K850 being the best performer were largely attributed to dataset size, with no significant correlation observed with the spectrum it can capture. Consequently, the conclusions drawn from previous studies when choosing the optimal filter for early disease detection have been rectified by addressing all limitations through the new balanced dataset.

A new balance multispectral dataset (https://drive.google.com/drive/folders/1RkqlyjP7M2tfjWbCRWOHSSktcClGCNqD?usp=sharing, accessed on 15 October 2023) of plants in the naturally complex background is a valuable asset for forthcoming research endeavours in early plant disease detection and advanced imaging techniques. Subsequent research efforts can capitalise on these insights by identifying diseases during their developing phases, augmenting the dataset dimensions, and fine-tuning the deep-learning models. Such progressions can elevate real-time plant disease identification using handheld devices, providing advantages for farmers, researchers, and the broader agricultural sector.

## Figures and Tables

**Table 1:** Visible and NIR distribution of wavelength ranges of filters used for image capturing.

Filter	Visible Portion	NIR Portion
K590	590–800 nm	800–1000 nm
K665	665–800 nm	800–1000 nm
K720	720–800 nm	800–1000 nm
K850	-	850–1000 nm
BlueIR	450–500 nm	800–1000 nm
Hot Mirror	400–800 nm	-

**Table 2:** Filters used for data collection, spectrum range, and sample images before and after preprocessing.

Filter		Wave Length Range (nm)	Sample	Sample after Prepossessing
K590	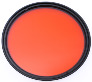	590–1000	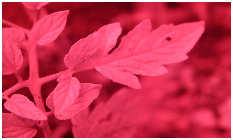	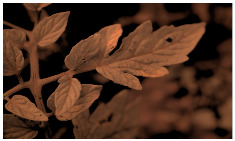
K665	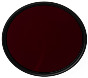	665–1000	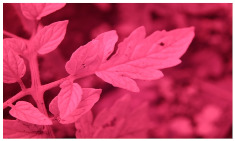	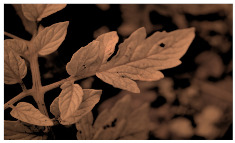
K720	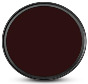	720–1000	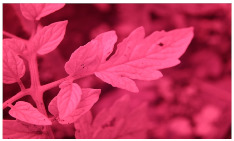	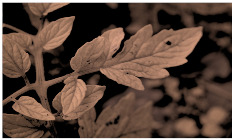
K850	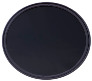	850–1000	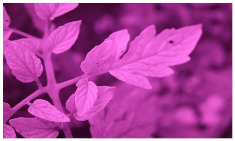	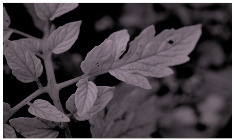
BlueIR	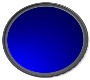	400–500 and 700–1000	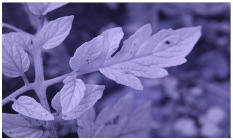	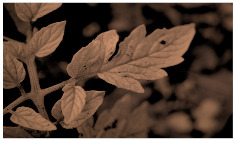
Hot Mirror	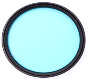	400–700	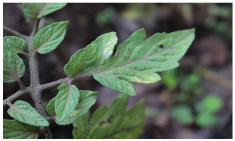	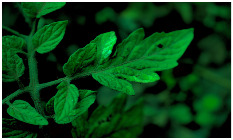

**Table 3:** The data composition for three distinct plant species captured using a single filter, categorised by plant type into “Healthy” and “Diseased” groups.

Plant	Healthy	Diseased
Papaya	60	49
Potato	80	105
Tomato	74	74

**Table 4:** Geometric transformation parameters used for training data augmentation.

Parameter	Value
rotation_range	25
shear_range	0.2
zoom_range	0.2
shear_range	0.2
zoom_range	0.2
horizontal_flip	True
vertical_flip	True

**Table 5:** Test Accuracy (%) of different sized Swin Transformer models for multiple filters on the unbalanced dataset in [19,20,21].

Model	K590	K850	BlueIR	Hot Mirror
Swin_tiny	75	79	85	79
Swin_small	71	80	86	75
Swin_base	71	81	83	84
Swin_large	75	80	86	84

**Table 6:** Test accuracy (%) of different sized CNN, ViT, Hybrid, and Swin Transformer models for multiple filters on the new balanced datasets.

Model	K590	K665	K720	K850	BlueIR	Hot Mirror
Xception	84	84	83	75	82	84
DenseNet121	84	83	88	78	81	83
ResNet50V2	74	75	76	78	70	80
ViT-S16	85	85	80	85	79	78
ViT-B16	84	84	84	83	82	83
ViT-L16	80	83	85	79	80	85
ViT_R26_S32	85	79	80	79	82	81
ViT_R50_L32	82	83	83	82	82	79
Swin_tiny	81	81	85	82	71	71
Swin_small	81	80	81	78	76	75
Swin_base	84	78	76	84	76	78
Swin_large	85	82	84	79	74	74

**Table 7:** Composition of healthy and diseased tomato data distribution in unbalanced and balanced datasets.

Plant Status	Unbalanced Dataset				New Dataset
	***K850***	***Hot Mirror***	***BlueIR***	***K590***	***All Six Filters***
Tomato Diseased	111	67	35	25	74
Tomato Healthy	111	144	114	22	74

**Table 8:** Precision of healthy and diseased tomato classes using various CNN, ViT, Hybrid, and Swin Transformer models on the newly balanced dataset.

Model	K590		K665		K720		K850		BlueIR		Hot Mirror	
	***Healthy***	***Diseased***	***Healthy***	***Diseased***	***Healthy***	***Diseased***	***Healthy***	***Diseased***	***Healthy***	***Diseased***	***Healthy***	***Diseased***
Xception	93	83	95	89	83	81	100	43	86	93	80	94
DenseNet-121	100	75	82	100	92	100	71	86	86	86	90	88
ResNet-50V2	86	75	77	78	83	100	71	71	71	79	80	75
ViT-S16	93	58	95	67	75	88	86	71	71	93	80	81
ViT-B16	100	75	95	78	100	81	100	86	79	100	100	88
ViT-L16	100	73	95	67	92	100	86	100	93	93	50	100
ViT_R26_S32	86	75	82	56	100	81	93	71	86	93	100	94
ViT_R50_L32	86	75	95	78	75	88	100	14	93	86	90	75
Swin_tiny	86	83	91	89	100	94	79	86	79	79	60	94
Swin_small	79	83	86	67	83	100	100	29	64	86	60	94
Swin_base	71	83	95	67	75	100	93	71	93	79	20	100
Swin_large	86	92	100	67	67	100	93	71	71	86	10	100

**Table 9:** Precision for healthy and diseased tomato classes using various CNN, ViT, Hybrid, and Swin Transformer models on the unbalanced dataset.

Model	K590		K850		BlueIR		Hot Mirror	
	***Healthy***	***Diseased***	***Healthy***	***Diseased***	***Healthy***	***Diseased***	***Healthy***	***Diseased***
Xception	50	60	95	67	83	33	100	92
DenseNet-121	80	60	95	81	100	33	90	85
ResNet-50V2	70	20	80	75	92	33	90	92
ViT-S16	57	25	93	84	100	38	100	100
ViT-B16	88	25	93	100	89	80	100	100
ViT-L16	71	50	93	84	100	70	94	100
ViT_R26_S32	77	40	96	78	83	40	97	100
ViT_R50_L32	77	20	100	72	83	40	97	78
Swin_tiny	80	40	76	73	96	50	88	100
Swin_small	60	60	82	82	92	100	84	94
Swin_base	80	0	88	77	92	100	94	94
Swin_large	80	60	82	77	88	100	94	100

**Table 10:** Recall of healthy and diseased tomato classes using various CNN, ViT, Hybrid, and Swin Transformer models on the newly balanced dataset.

Model	K590		K665		K720		K850		BlueIR		Hot Mirror	
	***Healthy***	***Diseased***	***Healthy***	***Diseased***	***Healthy***	***Diseased***	***Healthy***	***Diseased***	***Healthy***	***Diseased***	***Healthy***	***Diseased***
Xception	87	83	91	89	71	93	70	100	92	87	89	83
DenseNet-121	82	100	100	75	100	94	91	55	86	80	82	93
ResNet-50V2	80	82	85	58	100	89	62	36	77	69	62	86
ViT-S16	76	100	95	86	75	82	86	83	91	76	67	87
ViT-B16	82	100	91	88	75	100	88	100	100	82	83	100
ViT-L16	67	100	91	86	92	84	100	78	92	87	100	76
ViT_R26_S32	80	82	86	56	80	100	87	83	92	81	91	100
ViT_R50_L32	80	82	88	82	75	82	70	100	87	92	75	92
Swin_tiny	86	83	95	73	94	100	92	67	85	79	75	75
Swin_small	85	71	83	75	100	89	61	100	90	71	86	83
Swin_base	83	71	88	86	100	84	87	71	87	92	100	70
Swin_large	92	85	92	100	100	80	87	83	91	75	100	67

**Table 11:** Recall of healthy and diseased tomato classes using various CNN, ViT, Hybrid, and Swin Transformer models on the unbalanced dataset.

Model	K590		K850		BlueIR		Hot Mirror	
	***Healthy***	***Diseased***	***Healthy***	***Diseased***	***Healthy***	***Diseased***	***Healthy***	***Diseased***
Xception	71	38	67	82	71	64	97	100
DenseNet-121	67	60	82	96	69	100	93	100
ResNet-50V2	54	50	89	50	63	50	96	92
ViT-S16	57	25	81	94	79	75	97	100
ViT-B16	54	100	93	95	89	100	100	100
ViT-L16	83	50	81	94	86	100	77	100
ViT_R26_S32	67	50	89	93	79	40	100	90
ViT_R50_L32	71	33	86	93	75	33	97	100
Swin_tiny	62	40	81	100	83	100	100	100
Swin_small	86	43	82	82	92	33	100	94
Swin_base	73	0	83	100	92	100	100	100
Swin_large	80	75	82	89	92	50	100	100

## Data Availability

The data that support the findings of this study are available in Unbalance multispectral disease dataset (https://drive.google.com/drive/folders/1Ck9CKfru4SY9xknDSrWHqQM9EtXcjP_l?usp=drive_link, accessed on 15 October 2023.) and Balance multispectral disease dataset (https://drive.google.com/drive/folders/1RkqlyjP7M2tfjWbCRWOHSSktcClGCNqD?usp=sharing, accessed on 15 October 2023.).

## Appendix A

**Table A1:** F1 score for healthy and diseased tomato on the new balanced dataset using different sized CNN, ViT, Hybrid, and Swin Transformer models.

Model	K590		K665		K720		K850		BlueIR		Hot Mirror	
	***Healthy***	***Diseased***	***Healthy***	***Diseased***	***Healthy***	***Diseased***	***Healthy***	***Diseased***	***Healthy***	***Diseased***	***Healthy***	***Diseased***
Xception	91	83	93	89	77	87	82	60	89	90	94	88
DenseNet-121	90	86	90	86	96	97	80	67	86	83	86	90
ResNet-50V2	83	78	81	67	91	94	67	48	74	73	70	80
ViT-S16	84	74	95	85	75	85	86	77	80	84	73	84
ViT-B16	90	86	93	82	86	90	93	92	88	90	91	93
ViT-L16	80	50	93	75	92	91	92	88	89	90	67	86
viT_R26_S32	83	78	84	56	89	90	90	77	92	87	95	97
viT_R50_L32	83	78	91	88	75	85	82	25	90	89	82	83
Swin_tiny	86	83	93	80	96	97	85	75	81	79	67	83
Swin_small	81	77	84	71	91	94	76	44	75	77	71	88
Swin_base	77	77	91	75	86	91	90	71	90	85	33	82
Swin_large	89	88	96	80	100	80	90	77	80	80	18	80

**Table A2:** F1 score of healthy and diseased tomato on the unbalanced dataset using different sized CNN, ViT, Hybrid, and Swin Transformer models.

Model	K590		K850		BlueIR		Hot Mirror	
	***Healthy***	***Diseased***	***Healthy***	***Diseased***	***Healthy***	***Diseased***	***Healthy***	***Diseased***
Xception	59	46	78	73	77	44	98	96
DenseNet-121	73	60	88	88	81	50	92	92
ResNet-50V2	61	29	88	60	75	40	93	92
ViT-S16	57	25	87	89	88	50	99	100
ViT-B16	67	40	93	97	89	89	100	100
ViT-L16	77	50	87	89	92	82	85	100
viT_R26_S32	71	44	92	85	81	40	98	95
viT_R50_L32	74	25	93	81	79	36	97	88
Swin_tiny	70	40	79	84	89	67	93	100
Swin_small	71	50	82	82	92	50	92	94
Swin_base	76	0	86	87	92	100	97	97
Swin_large	80	67	82	83	90	67	97	100
